# *Helicobacter hepaticus* Induce Colitis in Male IL-10^−/−^ Mice Dependent by Cytolethal Distending Toxin B and *via* the Activation of Jak/Stat Signaling Pathway

**DOI:** 10.3389/fcimb.2021.616218

**Published:** 2021-03-12

**Authors:** Liqi Zhu, Chen Zhu, Shuyang Cao, Quan Zhang

**Affiliations:** ^1^ Institute of Comparative Medicine, College of Veterinary Medicine, Yangzhou University, Yangzhou, China; ^2^ Jiangsu Co-Innovation Center for Prevention and Control of Important Animal Infectious Diseases and Zoonoses, Yangzhou University, Yangzhou, China; ^3^ Affiliated Hospital of Yangzhou University, Yangzhou University, Yangzhou, China

**Keywords:** *Helicobacter hepaticus*, CdtB, colitis, Jak-Stat, IL-10^−/−^ mice

## Abstract

It has been well documented that cytolethal distending toxin (CDT) from *Helicobacter hepaticus* (*H. hepaticus*), *Campylobacter jejuni* (*C. jejuni*) and other Gram-negative intestinal pathogens is linked to the inflammatory bowel disease (IBD). However, the mechanisms underlying the progression of *H. hepaticus* induced colitis remains unclear. In this study, male B6.129P2-*IL10^tm1Cgn^*/J mice were infected by *H. hepaticus* and ΔCdtB *H. hepaticus* for 6, 12, 18, and 24 weeks. Histopathology, *H. hepaticus* colonization levels, expression of inflammatory cytokines, signaling pathways, and content of NO in proximal colon were examined. We found that Cytolethal distending toxin subunit B (CdtB) deletion had no influence on colonization ability of *H. hepaticus* in colon of B6.129P2-*IL10^tm1cgn^* ^/^J mice, and there was no significant difference in abundance of colonic *H. hepaticus* over infection duration. *H. hepaticus* aggravated rectocele and proximal colonic inflammation, especially at 24 WPI, while ΔCdtB *H. hepaticus* could not cause significant symptom. Furthermore, mRNA levels of *Il-6*, *Tnf-α*, *Il-1β*, and *iNOS* significantly increased in the proximal colon of *H. hepaticus*-infected mice compared to ΔCdtB *H. hepaticus* infected group from 12 WPI to 24 WPI. In addition, the elevated content of NO and activated *Stat3* and *Jak2* in colon were observed in *H. hepaticus* infected mice. These data demonstrated that CdtB promote colitis development in male B6.129P2-*IL10^tm1Cgn^*/J mice by induction of inflammatory response and activation of *Jak*-*Stat* signaling pathway.

## Introduction

*Helicobacter hepaticus* (*H. hepaticus*) belongs to *Proteobacteria*, *ϵ-proteobacteria*, *Heliobacterium*. *H. hepaticus*, the prototype of human enterohepatic helicobacters, infection is characterized by colitis (Xu et al., 2018), and induce colon cancer (Falsafi and Mahboubi, 2013) in susceptible mice strains. Immunodeficient mice infected by *H. hepaticus* are often used as animal models for inflammatory bowel disease (IBD) studies (Cahill et al., 1997). In general, *H. hepaticus* can cause IBD in IL-10 deficient mice, but is an enteric symbiotic bacterium in wild-type C57/BL6 mice. These results may be caused by the secretion of a high level anti-inflammatory cytokine by macrophages, which were activated by large polysaccharide secreted from *H. hepaticus* (Danne et al., 2017). But there are also contrary studies pointing out that *H. hepaticus* does not induce or potentiate colitis in IL-10 deficient Mice (Dieleman et al., 2000). Moreover, Mark et al. found that *H. hepaticus* do not induce typical colonic inflammation symptoms unless it co-infected with the potential probiotic *Lactobacillus reuteri* in Germ-free mice (Whary et al., 2011). Therefore, the mechanism of *H. hepaticus* inducing to inflammatory bowel disease is complicated, which needs to be further explored.

Cytolethal distending toxin (CDT), a known major virulence of *H. hepaticus*, is a type of AB_2_ toxin found in Gram-negative bacteria, including *Heliobacterium*, *Salmonella*, *Shigella*, *Campylobacter jejuni*, and *Escherichia coli*. The CdtA and CdtB subunits produce non-spherical amino acid extensions that interact with the CdtC subunit (Avenaud et al., 2004). Usually, the subunits CdtA and CdtC play the role of connecting transportation, transferring the active subunit CdtB into cells (Smith and Bayles, 2006). It has been reported that the biological functions of CdtB include phosphatase activity, blocking cell cycle, and eventually leading to cell death (Ge et al., 2008). CdtB is similar to members of the DNaseI family, which can bind to cations and contain DNA-catalyzed residues, but its hydrolysis activity to DNA is about 100 times lower than that of DNaseI (Dassanayake et al., 2005). Meanwhile, it activates the ATM-dependent DNA damage reaction in a myc-dependent pathway (Guerra et al., 2010). Many studies have found that CDT plays an important role in induction of IBD and carcinogenesis (Young et al., 2004; Ge et al., 2017). In previous studies, *H. hepaticus* infection has been shown significantly induced ileum and cecum *Ifn-γ* production and mucosal IgA secretion as well as down-regulated *Il-10* (Kullberg et al., 1998; Ge et al., 2005). In a recent study, Ge et al. demonstrated that CdtB could upregulate *Tnf-α* and *Il-6* expression and cause DNA double-strand break in cecal epithelial cells, as well as Stat3 activation, leading to precancerous lesions (Ge et al., 2017). However, the roles of CdtB of *H. hepaticus* induced colitis are still to be delineated.

Therefore, the purpose of this study was to investigate the effects of CdtB of *H. hepaticus* infection on inflammatory bowel disease by using a CdtB deleted *H. hepaticus* strain, and further clarify the mechanism of *H. hepaticus* infection induced colitis.

## Materials and Methods

### Mice

Female and male B6.129P2-*IL10^tm1Cgn^*/J mice (*Il10* ^−/−^ mice) were purchased from Jackson Laboratory (Stock No: 002251), all mice were bred and maintained in an accredited specific pathogen-free facility, and *in vivo* experiments were conducted in accordance with the China laboratory Act (2017) under a Project License [SYXK (su) 2007-0044] authorized by Jiangsu Provincial Science and Technology Department and approved by Institutional Animal Care and Use Committee (IACUC) of Yangzhou University. The mice were free of *Helicobacter* species as assessed by PCR as previously described (Qin et al., 2016).

### Bacterial Culture and Collection

CdtB mutant strain (ΔCdtB *H. hepaticus*) was constructed by Zhu et al. (2020), and conserved in our lab. In brief, the *CdtB* gene was replaced to Chloramphenicol resistant gene (*Cm*) by homologous recombination through electro-transforming the CdtA-Cm-CdtC fragment to *H. hepaticus* competent cell; then, the positive transformants were continuously subcultured on Brucella agar plates with chloramphenicol for five times. *H. hepaticus* 3B1 (ATCC 51449) and ΔCdtB *H. hepaticus* were cultured on Brucella agar plates (BD, USA) supplemented with 5% defibrinated sheep blood and antibiotics for 4–5 days under microaerobic conditions (85% N_2_, 10% CO_2_, 5% O_2_) at 37°C. Bacteria were harvested in PBS and used for oral infection when OD_600_ reading was 1 (Xu et al., 2018).

### Experimental Details

Approximately 60 5-week-old male mice were randomly divided into three groups. In order to promote the colonization of *H. hepaticus* in colon and rectum, the mice drank free water with gentamicin for 1 week (Kennedy et al., 2018), and then were infected with *H. hepaticus* and ΔCdtB *H. hepaticus* at the age of 6 weeks. The control group was inoculated with PBS. The mice were inoculated intragastrically with 200 μl of PBS containing 2 × 10^8^ *H. hepaticus* (Xu et al., 2018) every 2 days for a week, and the infection was confirmed by feces sampling. Five mice from each group were euthanized at 6, 12, 18, 24 WPI, respectively. Colon tissues were collected and stored at −80°C for analysis.

### DNA Extraction and *H. hepaticus* Quantitative PCR Analysis

The mice were fasted overnight before sacrificed. The proximal colon segments in the same position were selected, then the tissue and bacterial DNA was extracted according to manufacturer’s instructions using the TIANamp Bacteria DNA kit (Tiangen, Beijing). Abundance of *H. hepaticus* in colon was determined according to *HH1450* gene primers by qPCR according to the method established by Ge et al. (2005). In brief, to quantitative detect the copy number of *H. hepaticus*, the tissue DNA was diluted to 10 ng/μl and used as a template for quantitation PCR assay in the Applied Biosystems StepOne Real Time PCR System (ABI) using Universal SYBR Green master (ABI). Serial dilutions of *H. hepaticus* DNA, including 2 × 10^7^, 2 × 10^6^, 2 × 10^4^, 2 × 10^2^, 2 × 10^1^, and 2 fg, were used to generate a standard curve. 

### Histopathology

During necropsy, colon tissues of each animal were fixed in 4% paraformaldehyde for 24 h, and proximal, middle, and distal part embedded in paraffin for microscopy examination respectively. For histopathological analysis, tissue samples were cut into 4 μm sections, deparaffinized, and stained with hematoxylin and eosin (H&E). Histopathologic parameters were graded as described previously (Berg et al., 1996; Erdman et al., 2009). In brief, score from 0 to 4 was based on the following criteria: (grade 0) normal colonic mucosa without lymphocyte infiltration and disorganization; (grade 1) mild inflammation with mononuclear cell infiltrates in the lamina propria accompanied by minimal epithelial structure change; (grade 2) lesions tended to involve more of the colon than grade 1 lesions, or were more frequent; (grade 3) lesions involved a large area of the mucosa or were more frequent than grade 2 lesions; (grade 4) lesions usually involved most of the colonic section and were more severe than grade 3 lesions. Each sample was randomly selected for five slices, and given a score based on the criteria described above and the summation of these scores provided a total colonic disease score per mouse. The detailed pathological characteristics was referred to **Table 1**. The disease scores could range from 0 (no change in any segment) to a maximum of 20 (grade 4 lesions in all five segments).

**Table 1:** The criteria of Histologic analysis (Berg et al., 1996).

Histologic score (HS)	The criteria
0	No change from normal tissue;
1	One or a few multifocal mononuclear cell infiltrates in the lamina propria accompanied by minimal epithelial hyperplasia and slight to no depletion of mucus from goblet cells
2	Typical changes included several multifocal, mild inflammatory cell infiltrates in the lamina propria composed primarily of mononuclear cells with a few neutrophils. Mild epithelial hyperplasia and mucin depletion were also seen. Small epithelial erosions were occasionally present and inflammation rarely involved the submucosa;
3	Inflammation was moderate and often involved the submucosa but was rarely transmural. Inflammatory cells were a mixture of mononuclear cells as well as neutrophils, and crypt abscesses were sometimes observed. Moderate epithelial hyperplasia and mucin depletion were seen. Ulcers were occasionally observed;
4	nflammation was severe, including mononuclear cells and neutrophils, and was sometimes transmural. Epithelial hyperplasia was marked with crowding of epithelial cells in elongated glands. Few mucin-containing cells were seen. Crypt abscesses and ulcers were present.

### Cytokines Quantification by Real-Time RT-PCR

Total RNA was extracted from proximal colon tissue trituration in liquid nitrogen using Trizol (Roche, Germany). cDNA was synthesized from 1 μg total RNA using Prime Script RT reagent Kit with gDNA Eraser (Takara, Dalian). Transcript levels of colonic were amplified by qPCR using the Universal SYBR Green master and mRNA level of the GAPDH gene in each cDNA sample was measured and used for normalization. qPCR primers (*Il-1β*: TGATGAGAATGACCTCTTCT,CTTCTTCAAAGATGAAGGAAA; *Il-6*: TAGTCCTTCCTACCCCAATTTCC,TTGGTCCTTAGCCACTCCTTC; *Tnf-α*: AACTAGTGGTGCCAGCCGAT,CTTCACAGAGCAATGACTCC; *iNOS*: GTTGAAGACTGAGACTCTGG,GACTAGGCTACTCCGTGGA; MMP-9: TGGGGGGCAACTCGGC,GGAATGATCTAAGCCCAG; ZO-1: GGGGCCTACACTGATCAAGA,TGGAGATGAGGCTTCTGCTT; GAPDH: CCATCACCATCTTCCAGGAG,CCTGCTTCACCACCTTCTTG) were synthesized by Sangon Biotech (Shanghai) Co., Ltd. All samples were run in triplicate in the Applied Biosystems StepOne Real-Time PCR System. Relative expression levels were calculated using 2^-ΔΔCT^ method, as previously described (Livak and Schmittgen, 2001).

### ELISA

Total proteins were extracted from proximal colon tissue trituration in liquid nitrogen using IP Lysis Buffer (Thermo, USA) containing 1% PMSF and 1% phosphatase inhibitor cocktail. The samples were performed ultrasonic cracking at a power of 200 W for 5 s, then centrifuge at 12,000 rpm for 30 min at 4°C, and the *Il-6* content from supernatant was detected with the Mouse IL-6 ELISA kit (Boster, Wuhan).

### Western Blot Analysis

Total proteins were extracted from proximal colon tissue trituration in liquid nitrogen using RIPA lysis buffer (Thermo, USA) containing 1% PMSF and 1% phosphatase inhibitor cocktail. The samples were performed ultrasonic cracking at a power of 200 W for 5 s, then centrifuge at 12,000 rpm for 15 min at 4°C. The amount of protein was determined by BCA kit, and 40 μg of total proteins was loaded into 10% SDS-PAGE. After electrophoresis, proteins were transferred to a 0.22 μm PVDF membrane at 300 mA for 1.5 h, 5% skim milk was used for blocking at room temperature for 2 h. The membranes were incubated with primary antibody overnight at 4°C, including p-*Stat3*, *Stat3*, p-*Jak2*, *Jak2*, GAPDH (1:1,000, Cell Signaling Technology), and then incubated with secondary antibody conjugated to horseradish peroxidase (1:3,000, Cell Signaling Technology) at room temperature for 1 h. The proteins were visualized by Amersham ECL Select Western Blotting Detection Reagent.

### Statistical Analysis

Statistical analysis was performed using SPSS 18.0 software. Pathological scores were analyzed using a Mann-Whitney nonparametric *U* test. *H. hepaticus* colonization levels, cytokines, ELISA, and protein expression were analyzed using unpaired two-tailed Student’s *t* test. Figures were drawn using GraphPad 8.0 software, * refers to the *P* value when compared with the control group, * for *P* < 0.05, ** for *P* < 0.01, and *** for *P* < 0.001. 

## Results

### Lack of CdtB Did Not Affect the Colonization Efficiency of ΔCdtB *H. hepaticus*

**Figure 1:**
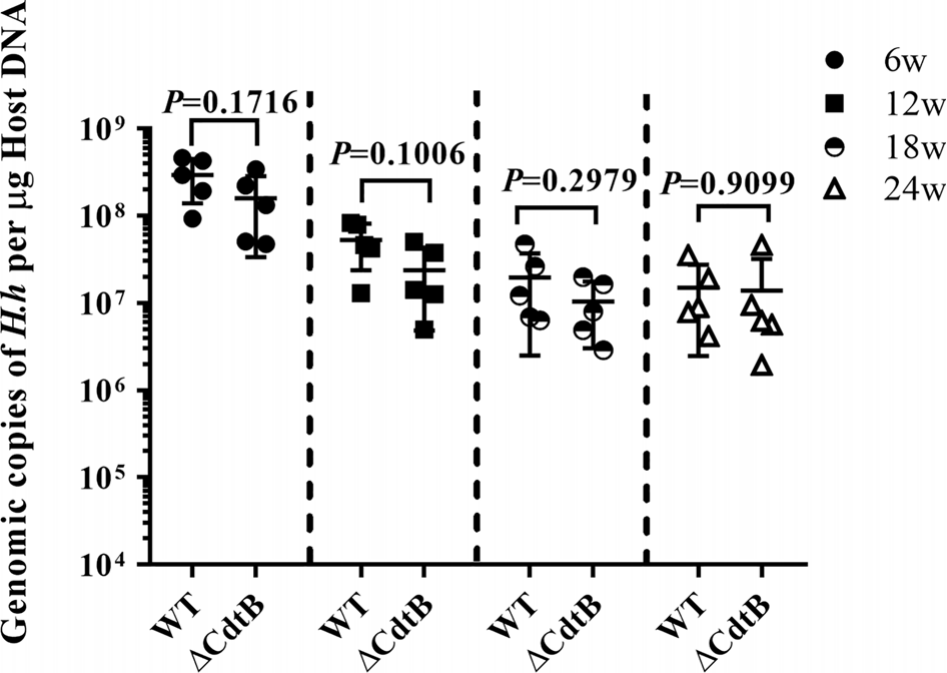
The absence of CdtB did not affect the colonic colonization of *H. hepaticus* in mice. The levels of *H. hepaticus* in the respective samples were expressed as its genomic copies per μg of mouse colon DNA. Data are expressed as the means ± SEM (n = 5/group). Statistics were analyzed using unpaired two-tailed Student’s *t* test.

It was reported that the ΔCdtB *H. hepaticus* lose their colonization efficiency over time in outbred Swiss Webster mice, inbred A/JCr mice and *Il-10* ^−/−^ mice (C57BL/6 background) (Ge et al., 2005; Pratt et al., 2006; Ge et al., 2007). We also examined the abundance of wild type *H. hepaticus* and CdtB mutant *H. hepaticus* in B6.129P2-*IL10^tm1Cgn^*/J mice of this study. Quantitative PCR was used to obtain the standard curve of *H. hepaticus*, and the abundance of two strains in the colon of *Il10* ^−/−^ mice was detected at necropsy. The results revealed that *H. hepaticus* and ΔCdtB *H. hepaticus* could be able to colonize in the colon of *Il10* ^−/−^ mice over the infection time (**Figure 1**), and there was no significant difference between *H. hepaticus* and ΔCdtB *H. hepaticus* colonization levels. 

### *H. hepaticus* Infection Induced Colitis in *Il-10* ^−/−^ Mice

**Figure 2:**
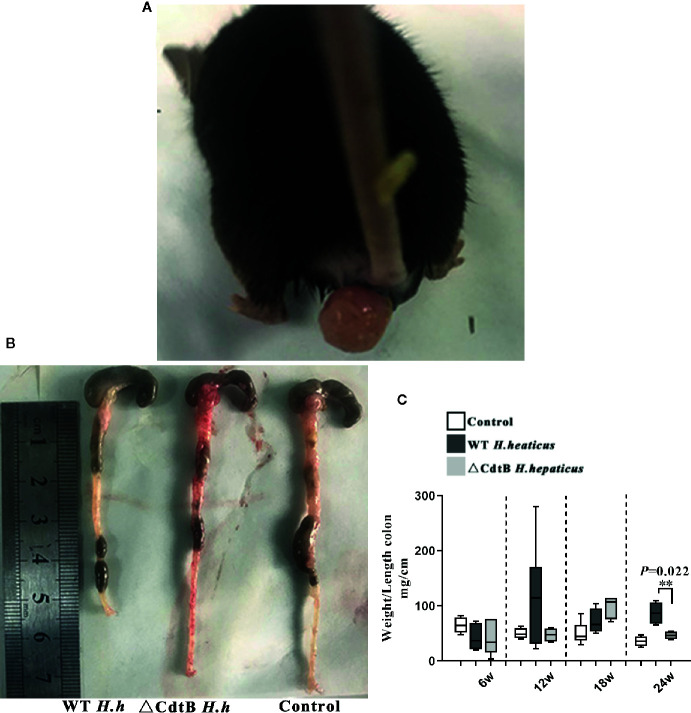
Deletion of CdtB alleviated colitis symptoms induced by (*H*) *hepaticus* infection. **(A)** Rectocele of mice in *H. hepaticus* group; **(B)** colonic segment of the experimental groups at 24 w; **(C)** weight/length ratio of the colon; Data are expressed as the mean ± SEM (n = 5/group). **represents *P* values <0.01 compared with the control group.

The weight/length ratio of the colon is an important parameter in the evaluation of intestinal inflammation. The results showed that there was no difference between the groups at 6 WPI, while anorectal prolapse was found in WT *H. hepaticus* infected mice (**Figure 2A**), and the weight/length ratio of mice infected by *H. hepaticus* was of significant difference compared with ΔCdtB *H. hepaticus* group at 24 WPI (*P* = 0.022) (**Figures 2B, C**). In addition, the symptoms of rectocele and watery stools in *H. hepaticus* group gradually worsened compared with ΔCdtB *H. hepaticus* group, suggesting that CdtB is an important virulence factor of *H. hepaticus* induced colitis in *Il-10* ^−/−^ mice.

### CdtB Promoted the Severity of Colitis Over the Infection

**Figure 3:**
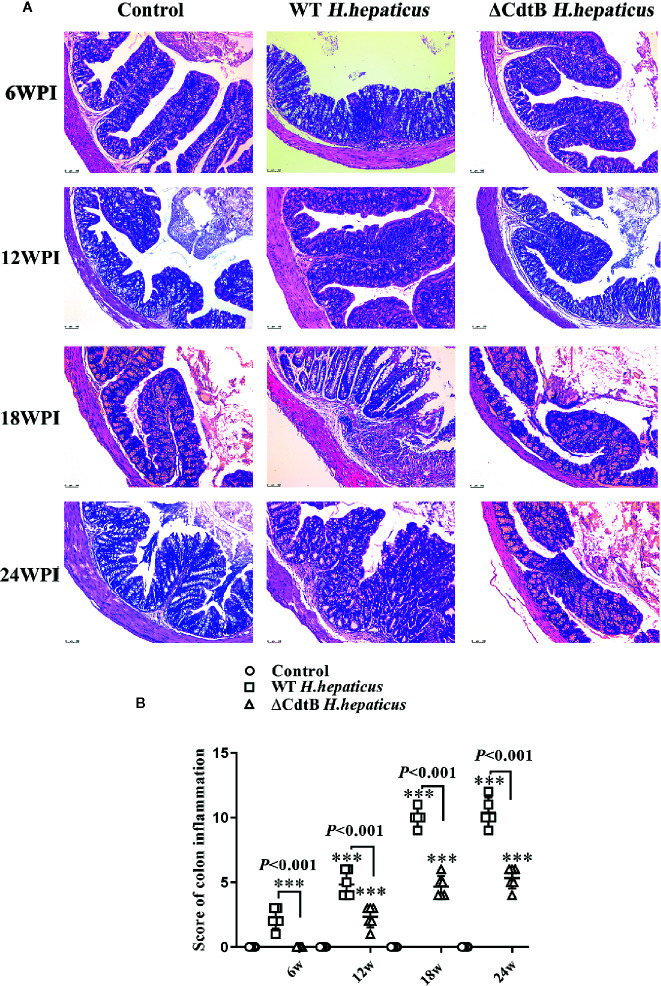
The colonic mucosa of colitic mice was attenuated by CdtB mutant. **(A)** Sections of the colonic mucosa were stained with hematoxylin and eosin (×100). At 6 WPI, the control colonic mucosa was integrated, crypts and goblet cells were evenly distributed, and a few scattered lymphocytes were found in submucosa; but WT *H. hepaticus* induced mild mononuclear cell infiltrates in lamina propria accompanied by slight depletion of mucus from goblet cells. At 12 WPI, ΔCdtB *H. hepaticus* induced a few multifocal mononuclear cell infiltrates in the lamina propria accompanied by slight to no depletion of mucus from goblet cells; while, WT *H. hepaticus* induced mild inflammatory cell infiltrates in the lamina propria accompanied by depletion of mucus from goblet cells. During the 18–24 WPI, the change of colonic symptoms caused by ΔCdtB *H. hepaticus* was not obvious; but WT *H. hepaticus* induced progressive colitic symptoms, including moderate lamina propria or submucosa inflammation, mucin depletion, crypts abscesses or hyperplasia and enlargement, etc. **(B)** Microscopic scores were assigned to the different groups according to the criteria described (Erdman et al., 2009). Data are expressed as the means ± SEM (n = 5/group). ***P < 0.001 when compared with the control group.

Histopathological examination was performed to characterize the role of CdtB in *H. hepaticus* infection induced colitis in *Il-10* ^−/−^ mice. Pathological changes included inflammation, edema, epithelial defect, atrophy, hyperplasia, and dysplasia. As shown in **Figure 3**, there was no inflammation observed in *H. hepaticus* infected mice at 6 WPI, but *H. hepaticus* infection promoted inflammation and edema, with mild epithelial defect, atrophy, hyperplasia, and dysplasia at 12 WPI. While at 18 WPI, proximal bowel inflammation score of *H. hepaticus* infected mice was significantly higher than that of ΔCdtB *H. hepaticus* infected group and severe inflammation were presented with inflammatory cells infiltrating the lamina propria and submucosa (*P* < 0.05). Furthermore, fibrin exudation in the intestinal glands and mucous epithelial exfoliation were also found in *H. hepaticus* infected mice. However, no fibrosis was observed (data not shown). By contrast, ΔCdtB *H. hepaticus* infected group developed into mild inflammation without typical pathological changes. Taken together, these data suggest that CdtB has a significant effect on *H. hepaticus*-induced inflammation. Interestingly, the proximal colon lesion was more obvious in the *H. hepaticus* infected group. Therefore, subsequent studies focused on the proximal colon.

### CdtB Induce Proinflammatory Response in Proximal Colon

**Figure 4:**
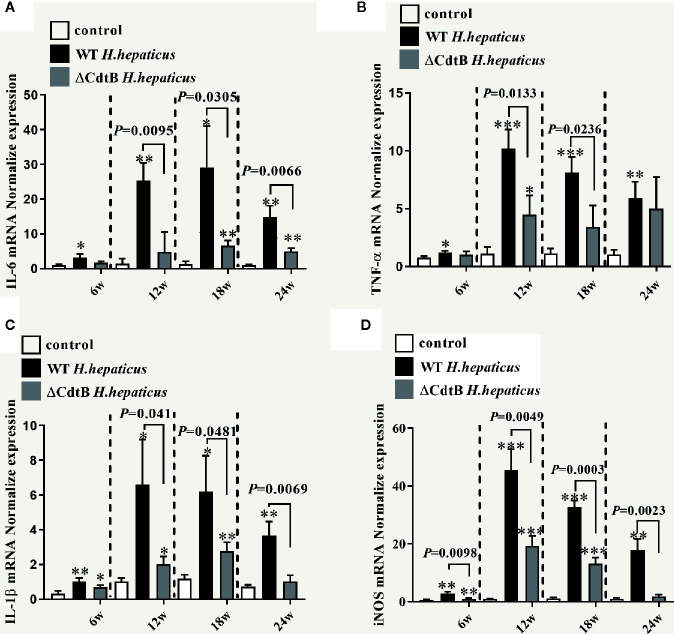
*H. hepaticus* infection induced the expression of pro-inflammatory cytokines. Colonic gene expression of pro-inflammatory cytokines **(A)** *Il-6*, **(B)** *Tnf-α*, **(C)** *Il-1β*, and **(D)** *iNOS* was analyzed by real-time qPCR and normalized with the housekeeping gene GAPDH. Data are expressed as the means ± SEM (n = 5/group). * refers to the *P* value when compared with the control group, *P < 0.05, **P < 0.01, and ***P < 0.001.

*Tnf-α*, *Il-1β*, *Il-6*, and *iNOS* are crucial in the pathogenesis of colitis (Luo and Zhang, 2017). In addition, *iNOS* has also been shown to be associated with the occurrence of intestinal inflammation because the increase of *iNOS* expression in inflammatory regions is related to histological inflammatory parameters (Palatka et al., 2005). As shown in **Figure 4**, the results showed that only *iNOS* have differences between infected groups at 6 WPI. While *H. hepaticus* significantly increased the *Il-1β* (*P* = 0.041), *Il-6* (P = 0.0095), *Tnf-α* (*P* = 0.0133), *iNOS* mRNA transcription level (*P* = 0.0049) at 12 WPI. Similarly, at 18 WPI and 24 WPI, CdtB promoted cytokine production detected by qPCR (excluding the *Tnf-α* at 24 WPI).

**Figure 5:**
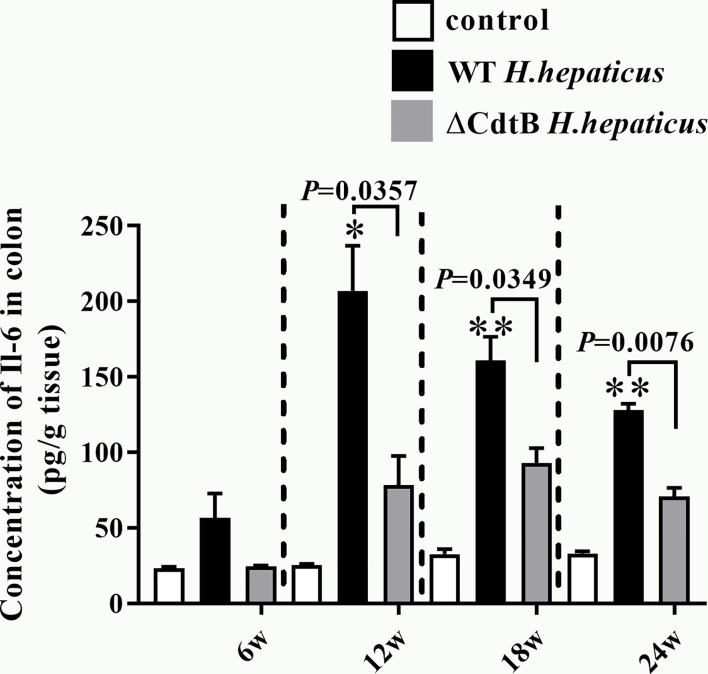
*H. hepaticus* infection induced the protein expression of *Il-6* in proximal colon. The cytokine levels of proximal colon tissue were expressed as the concentration in pg/g. Data are expressed as the means ± SEM (n = 5/group). *refers to the *P* value when compared with the control group, *for *P* < 0.05, **for *P* < 0.01.

To better determine *Il-6* protein levels, the expression of *Il-6* in the proximal colon was detected by ELISA. Consistent with the results obtained by qPCR, there was no significant difference between the infected groups at 6 WPI (**Figure 5**). However, *H. hepaticus* infection significantly increased the *Il-6* protein expression levels compared to ΔCdtB *H. hepaticus* at 12 WPI (*P* = 0.0357). Semblable results appeared at 18 WPI (*P* = 0.0349) and 24 WPI (*P* = 0.0076), but *Il-6* protein levels of *H. hepaticus* group decline from 12 WPI to 24 WPI. This may due to the immune tolerance of the colon. Taken together, the data showed that CdtB up-regulated *Il-6* protein expression during infection.

Tight Junctions and extracellular matrix are important for intestinal barrier stability (Bollyky et al., 2011; Parikh et al., 2019). *Zo-1* can prevent bacterial translocation, improve intestinal permeability (Arrieta et al., 2006), and maintain epithelial integrity to reduce colitis (Macdonald and Monteleone, 2005). *Mmp-9* is a family of proteolytic zinc enzyme and calcium-dependent structural proteins that degrade the extracellular matrix and are involved in the pathogenesis of IBD and experimental colitis (Koelink et al., 2014). However, there was no significant change in *Mmp-9* and *Zo-1* associated with colitis during the experiment probably for immune protection of the colon (data not shown). These data suggest that CdtB enhances the production of proinflammatory cytokines of *Tnf-α*, *Il-1β*, *Il-6*, and *iNOS* during infection.

### CdtB Increase NO Content in Proximal Colon

**Figure 6:**
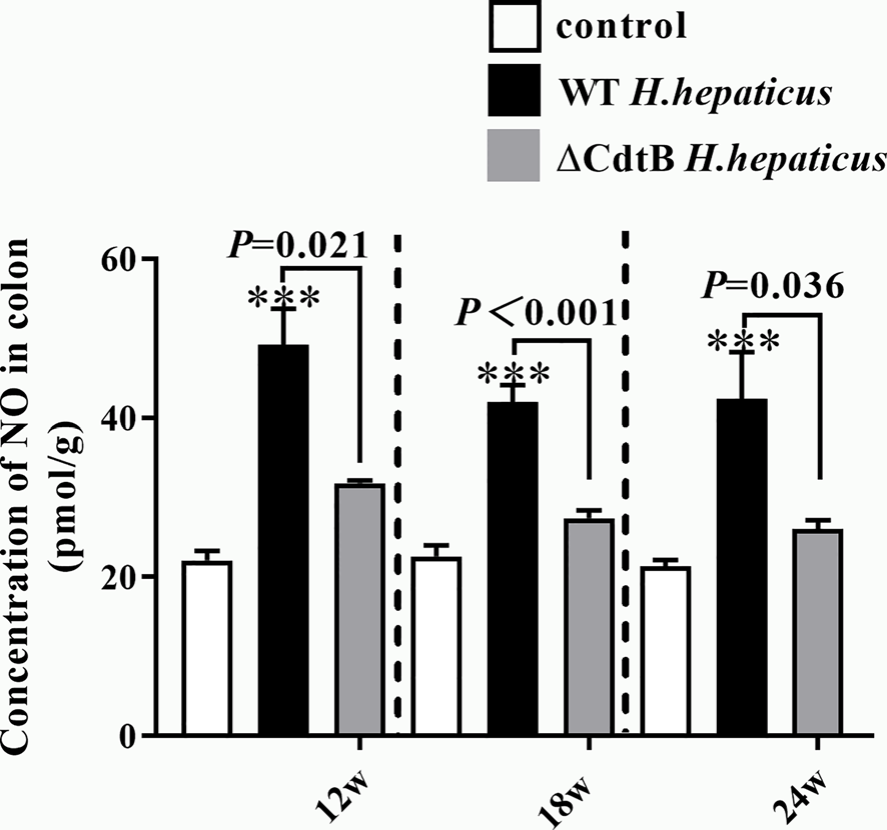
*H. hepaticus* infection induced the content of NO. The content of NO in colon tissue was expressed as the concentration in pmol/g. Data are expressed as the means ± SEM (n = 5/group). ***means *P* < 0.001 as compared to Control.

The content of NO was detected due to the differences in *iNOS between H. hepatius* infected and ΔCdtB *H. hepaticus* infected mice. It has been proposed that the increase of NO produced by *iNOS* will react with superoxide to form peroxynitrite, resulting in harmful changes in protein structure and function (Rachmilewitz et al., 1995). In the current study, from 12 WPI to 24 WPI, NO content in the gut was both significantly increased in ΔCdtB and WT *H. hepaticus* infection groups, especially raised much higher in WT *H. hepaticus* infection group (**Figure 6**). While, there was no difference between *H. hepaticus* groups at each time point (**Figure 6**). The data showed that CdtB caused the body to produce more NO during infection, which may link to CdtB ability of DNA damage and proto-oncogene expression.

### CdtB Promoted the Activation of *Jak*-*Stat* Signaling Pathways

**Figure 7:**
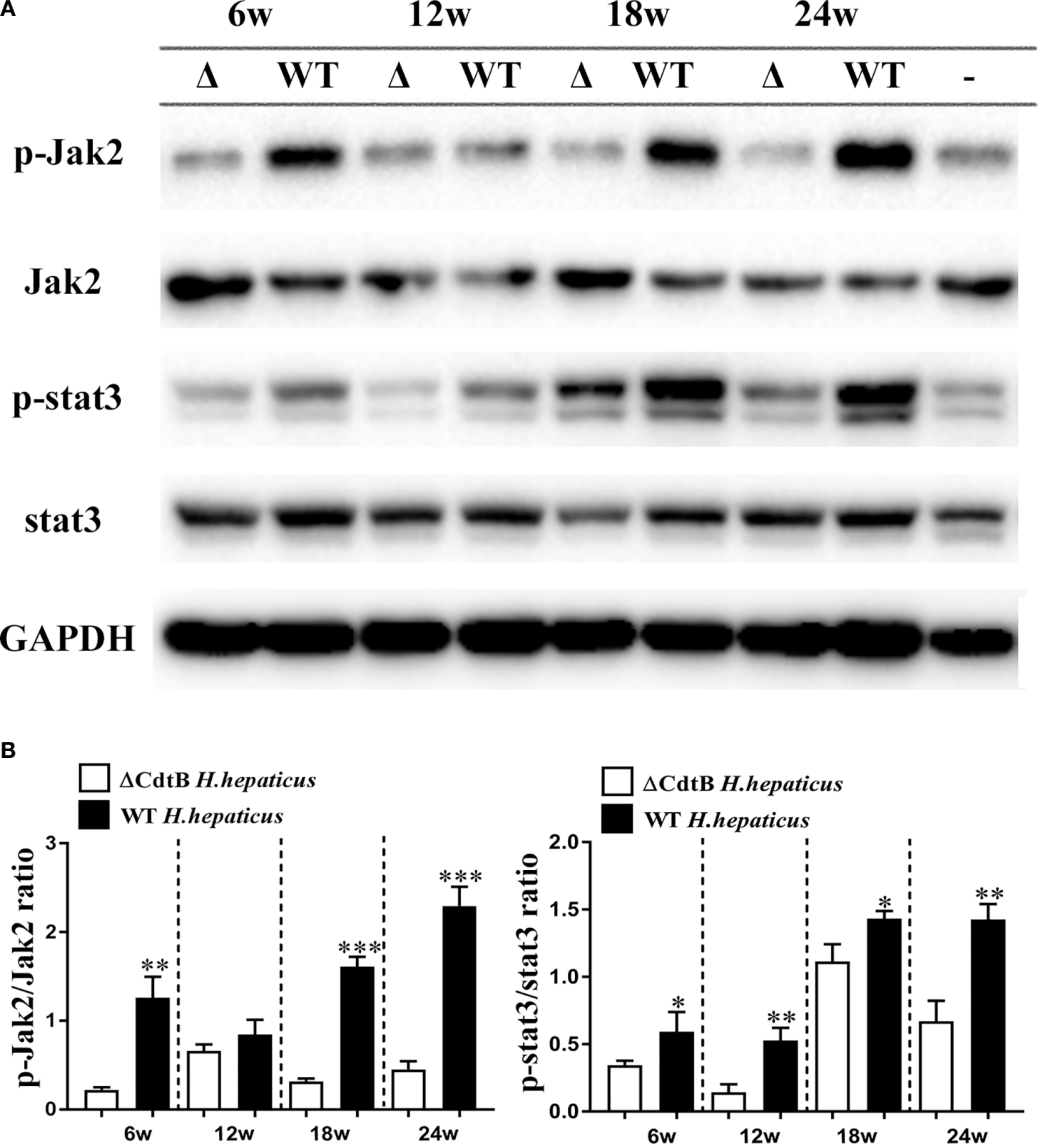
*H. hepaticus* promoted activation of *Jak2* and *Stat3*. **(A)** Western blotting and densitometric analysis of p-*Jak2*, *Jak2*, p-*Stat3*, *Stat3*, and GAPDH; WT represents wild type *H. hepaticus*, Δ represents ΔCdtB *H. hepaticus*, and – represents control sample of 24 WPI. **(B)** The Statistical data are expressed as the mean SD (n = 5). *P < 0.05 as compared to ΔCdtB *H. hepaticus*, **P < 0.01 as compared to ΔCdtB *H. hepaticus*, ***P < 0.001 as compared to ΔCdtB *H. hepaticus*.

The *Jak*-*Stat* signaling pathway plays a pivotal role in the pathogenesis of IBD, such as controlling and maintaining the development and differentiation of mucosal immune system and T help cells (Coskun et al., 2013). In order to understand whether IBD caused by *H. hepaticus* infection is related to this pathway, the protein extracted from the colonic epithelium was detected by immunoblotting (**Figure 7A**). The statistical result (**Figure 7B**) showed that only the phosphorylation of *Jak2* (p-*Jak2*) was significantly increased at 6 WPI in *H. hepaticus* groups compared to ΔCdtB *H. hepaticus*; while, at 18 and 24 WPI, both p-*Jak2* and p-*Stat3* was significantly increased in *H. hepaticus* groups compared to ΔCdtB *H. hepaticus*. These data suggest that CdtB promote the JAK-STAT signaling pathway activation during *H. hepaticus* infection, and plays an important role in *H. hepaticus*-induced colitis.

## Discussion

It has been reported that CdtB is essential for the long-term colonization of *H. hepaticus* in the gut of Swiss Webster and A/JCr mice with complete immune system (Ge et al., 2005; Ge et al., 2007). In contrast, the ΔCdtB *H. hepaticus* used in this study and *H. hepaticus* 3B1 had similar levels of colonization in B6.129P2-*IL10^tm1Cgn^/*J mice, which are consistent with the results obtained by Ge (Ge et al., 2017).This difference may be due to mouse strains that have different immune tolerance to *H. hepaticus* infection. In our study, *H. hepaticus* did not induce the expected degree of colitis. In the initial experiment, the common infection procedure failed to infect *Il-10* ^−/−^ mice from the Jackson Laboratory. Interestingly, when we hybridized male *Il-10* ^−/−^ mice from Jackson Laboratory with female wild type B6 mice from Model Animal Research Center of Nanjing University, then hybridized female *Il10* ^+/−^ heterozygote with male *Il-10* ^−/−^ mice, the homozygote of offspring obtained were highly susceptible to *H. hepaticus*, which suggested the important role of gut flora in the process of *H. hepaticus* infection. Pretreatment of mice with gentamicin drinking water can eliminate part of the normal microflora of the gut, and increase the colonization of *H. hepaticus* in the cecum and colon. Compared with other reports, the continuous infection at 24 WPI of mice in this study did not induce severe colitis, which may be due to the fact that some bacterial which was not completely eliminated by gentamicin protected the gut from *H. hepaticus* colonization. To explore possible reasons, we performed fecal microbiota transplantation experiment in the next stage (data not shown), and high-throughput sequencing related to gut flora also requires further research.

In this study, CdtB up-regulated the transcription of *Il-6* and induced *Jak2*/*Stat3* signaling pathways. Colitis in *Jak3* knockout mice is thought to be caused by a defect in the intestinal barrier. *Jak3* knockout mouse model showing *Il-2*, *Il-4*, *Il-7*, *Il-9*, and *Il-15* conduction defects, elevated levels of *Il-6* and *Il-17a* and severe immunodeficiency, defects in barrier function lead to Ulcerative colitis (UC) (Baird et al., 1998; Mishra et al., 2013). It is currently believed that the *Il-6*/*Stat3* pathway promotes the apoptosis resistance of intestinal T lymphocytes by inducing the activation of the downstream anti-apoptotic genes *Bcl-2* and *Bcl-xl*, resulting in the massive release of inflammatory factors and the continued destruction of the organization (Carey et al., 2008). Suzuki et al. (2001) knocked down the *Il-6* gene in mice induced by dextran sodium sulfate (DSS), the severity of colitis and the level of activated Stat3 were significantly reduced, and *Il-22* activated *Stat3* to promote intestinal epithelium cellular (IEC) homeostasis and tissue healing indicate that the activation of *Stat3* is an important factor in the persistence of colon inflammation. In UC, the differentiation of lamina propria T cells into Th2 cells is mediated by *Il-4*, which produces Th2 type cytokines *Il-4*, *Il-5*, and *Il-13*, and naive T cells are sent out through *Jak1–Jak3* Signal and activate Stat6. Barrett et al. (2008) found that the rs10758669 risk site associated with UC is located in the *Jak2* region. In the pattern-recognition receptor (PRR)–induced UC, its anti-inflammatory cytokines gradually decreased with the decrease of *Jak2* expression. PRR-induced reduction of inflammatory factors such as *Il-10*, *Il-4*, *Il-22* depends on *Jak* to regulate inflammation and induce UC (Hedl et al., 2016).

Colitis can cause massive infiltration of immune cells in the inflamed tissues of the colon, release a variety of pro-inflammatory cytokines, and produce high levels of NO and lipid peroxidation (Araujo et al., 2017). Proinflammatory cytokines are the basic regulators of colitis development (Araujo et al., 2017), and imbalance between intestinal mucosal immunity can damage the colonic mucosa and affect intestinal homeostasis (Zhang et al., 2017). CdtB can significantly stimulate cells to produce NO, which may be related to the induction of DNA damage and the upregulation of proto-oncogene expression. In this study, it was observed that CdtB can up-regulate *Jak2*/*Stat3* phosphorylation, suggesting that the promotion of *Jak*/*Stat* pathway may be involved in the effect of *H. hepaticus* CdtB on colitis, which is related to the expression of proinflammatory cytokine-related genes. The key role of *Stat3* signal transduction in inflammation has been demonstrated in colitis-related mouse models (Bollrath et al., 2009; Grivennikov et al., 2009). Infection of mice with *H. hepaticus* can cause colitis, including rectocele, watery stool, intestinal epithelial damage, inflammatory cell infiltration, and reduction in colon length. Studies have shown that CdtB can induce mammalian cells cytopathy through a series of cellular reactions (Ge et al., 2008). Although there are many reports on clarifying CdtB-induced cytopathy, the pathogenic mechanism of CdtB in bacterial infected animal has not been fully clarified. This study demonstrated that CdtB promoted the progression of inflammation within 24 weeks in B6.129P2-*IL10^tm1Cgn^*/J mice infected with *H. hepaticus*, and up-regulated the transcription of *Il-6* and *Tnf-α* in the proximal colon during infection. In addition, the mutant of CdtB reduced the ability of *H. hepaticus* to activate *Jak2* and *Stat3* signaling pathways, but did not affect the colonization efficiency of *H. hepaticus*. Taken together, these data suggest that CdtB may enhance *H. hepaticus*’ inflammatory ability by activating the *Il-6*/*Jak2*/*Stat3* signaling pathway. In view of the role of CdtB in promoting the occurrence of gut inflammation, the role of bacterial CDT on the pathogenicity of intestinal is worthy of further study.

## Data Availability Statement

The original contributions presented in the study are included in the article/supplementary material. Further inquiries can be directed to the corresponding author.

## Ethics Statement

The animal study was reviewed and approved by the Institutional Animal Care and Use Committee (IACUC) of Yangzhou University.

## Author Contributions

LZ processed data and wrote manuscript. CZ constructed the CdtB deficient *H. hepaticus* strain and carried out animal experiments. SC carried out the histological and immunoblotting experiments. QZ designed the experimental scheme and provided funds. All authors contributed to the article and approved the submitted version.

## Funding

This work was supported by the National Key Research and Development Program of China (2017YFD0502303, 2017YFD0501605), the Key Research and Development Program of Jiangsu Province (BE2020674), the Priority Academic Program Development of Jiangsu Higher Education Institutions (PAPD), High-end Talent Support Program of Yangzhou University and the Young and Middle-Aged Academic Leaders Plan of Yangzhou University.

## Conflict of Interest

The authors declare that the research was conducted in the absence of any commercial or financial relationships that could be construed as a potential conflict of interest.
